# Correlations of Disc Tissue Pathological Changes With Pfirrmann Grade in Patients With Disc Herniation Treated With Microdiscectomy

**DOI:** 10.7759/cureus.37913

**Published:** 2023-04-21

**Authors:** Mahmut Ozden, Zuhal K Silav

**Affiliations:** 1 Neurosurgery, Memorial Bahcelievler Hospital, Istanbul, TUR; 2 Pathology, Sadi Konuk Education and Research Hospital, Bakirkoy, TUR

**Keywords:** brachyury, p53, macrophage, cd68, cd34, disc hernia

## Abstract

Background: To reveal whether pathological disc changes (vascularization, inflammation, disc aging and senescence as assessed with immunohistopathological CD34, CD68, brachyury and P53 staining densities respectively) are associated with the extent of disease (Pfirrmann grade) and lumbar radicular pain in patients with lumbar disc herniation. We selectively included a homogenous group of 32 patients (16 males and 16 females) with single-level sequestered discs who had disease stages between Pfirrmann grades I to IV and excluded patients with the complete collapse of the disc space to determine histopathological correlations of the disease more precisely.

Materials and methods: Pathological assessments were performed on surgically excised disc specimens stored in a -80°C refrigerator. Preoperative and postoperative pain intensities were determined with visual analog scales (VASs). Pfirrmann disc degeneration grades were determined on routine T2-weighted magnetic resonance imaging (MRI).

Results: Stainings were especially observed with CD34 and CD68, which positively correlated with each other and Pfirrmann grading but not with VAS scores or patients' age. Weak nuclear staining with brachyury was observed in 50% of patients and did not correlate with disease features. Focal weak staining with P53 was only seen in the disc specimen of two patients.

Conclusions: In the pathogenesis of disc disease, inflammation may trigger angiogenesis. The subsequent aberrant increase of oxygen perfusion in the disc cartilage may cause further damage, as the disc tissue is adapted to hypoxia. This vicious cycle of inflammation and angiogenesis may be a future innovative therapeutic target for chronic degenerative disc disease.

## Introduction

The most common cause of radicular pain is lumbar disc herniation with a lifetime prevalence of about 10% [1]. One of the most likely causes of this problem is intervertebral disc degeneration and according to some studies, this degeneration is the key alteration that causes low back pain [1,2]. Hence, one would expect that the intensity of pain in patients with disc herniation would directly correlate with pathological disc alterations. The conflicting results regarding the correlation between symptoms of pain and disease extent in disc herniation need to be investigated with further studies. One of the aims of this current study is to assess the association of pain scores with Pfirrmann grades of disc disease. A healthy intervertebral disc consists of an inner, soft, and hydrated nucleus pulposus and an outer collagenous annulus fibrosus. Intervertebral discs exert very low cellularity (0.25%-0.5%) and constitute chondrocyte‑like cells in the nucleus pulposus, apparently necrotic cells in the inner annulus, and spindly tendon-like cells in the outer annulus. Nucleus pulposus cells and cells residing in the inner annulus fibrosis are deprived of sufficient blood supply; hence, oxygen levels in these areas are very low. After the early years of life, vascular invasion into the inner layers of the annulus fibrosus and cartilaginous endplates is generally associated with injury or disruption of disc tissue, irrespective of age [3]. In adults, the intervertebral disc is the largest avascular organ of the human body; therefore, the nucleus pulposus is isolated from the immune system (blood-nucleus pulposus [BNP] barrier) [4].

Also, the immunomodifying molecules released by disc cartilage cells and sparse vessels inhibit immunocyte infiltration and inflammatory cytokines. For instance, Fas ligand (FasL), a molecule inducing CD8+ T lymphocyte and macrophage apoptosis, widely expressed in immune sanctuary sites, is also expressed in human nucleus pulposus [4]. Hence, it may act as an immune barrier hindering microvascular infiltration and tissue invasion of immunocytes in a physiological state. Further, an annulus fibrosis-conditioned medium is shown to reduce the release of pro-inflammatory molecules including IL-1β, TNF-α, and MCP-1 [4]. Lastly, there also exists some data that certain miRNAs and exosomes may contribute to the immunosuppressive microenvironment of intervertebral discs. Hence, the intervertebral disc is an immune-privileged organ due to the BNP, which if damaged, will lead to disc degeneration, radiculopathy, and also a seemingly paradoxical regression of the herniated disc [4]. Kwon et al demonstrated that hypoxia primes cartilaginous disc cells for the higher release of matrix-degrading enzymes (matrix metalloproteases [MMPs]) and angiogenesis-stimulating vascular endothelial growth factor (VEGF) in the presence of inflammatory cytokines [2]. Peculiarly, an enhancement of oxygen concentration after disc degeneration could even act pathologically, as disc tissue cells normally live in hypoxia as a result of adaptive mechanisms such as anaerobic glycolysis [2]. Therefore, in this study, we aimed to evaluate angiogenesis (with endothelial cell marker CD34) and inflammation (with macrophage cell marker CD68) in correlation with the extent of disc disease as assessed by Pfirrmann's grading. We also assessed whether CD34 and CD68 staining densities correlated with the age of patients with disc herniation, their presurgical pain intensities, and their postsurgical healing in terms of pain levels. Although several studies regarding these aspects exist, conflicting results were obtained due to several reasons including that CD68+ macrophages are involved in disc degeneration and spontaneous regression of disc disease via removal of the extruded pathological disc tissues [5,6]. Hence, further studies conducted in properly selected patient populations are still needed. We also assessed immunoexpressions of P53 and brachyury in surgically excised herniated human disc tissues to reveal the senescence of disc tissues in disc disease. The particular reasons for choosing these markers are explained in the discussion section in a more detailed manner.

## Materials and methods

Study focus and ethical issues

This study was conducted to analyze whether pathological disc changes (vascularization, macrophage infiltration, and senescence as assessed with CD34, CD68, brachyury, and P53 staining densities respectively) are associated with the extent of disease (Pfirrmann grade) and lumbar radicular pain in patients with single sequestered disc disease. Thirty-two patients (16 males, 16 females) with lumbar microdiscectomy operations were selected to participate in the study after obtaining informed consent forms. The procedures followed were in accordance with the ethical standards of the institutional and responsible regional committee on human experimentation and with the Helsinki Declaration of 1975, as revised in 2000. Ethical approval was obtained from the local ethical committee of Memorial Bahcelievler Hospital (approval number: 66; Istanbul, Turkey).

Inclusion and exclusion criteria

The study included patients whose radiological features of single-level lumbar disc herniation correlated with the intensity of lumbar radicular pain. All patients had radicular pain without spinal stenosis observed on magnetic resonance imaging (MRI). The exclusion criteria other than described above were as follows: diffuse neurological deficits (cauda equina syndrome), lumbar spine pathologies of other etiologies including infections, previous fractures, primary spinal tumors and metastases, advanced degenerative diseases of vertebral bones, spinal column malformations and other neurologic, osteologic, and muscular diseases associated with pain.

Clinical assessment

Demographic factors (i.e., age, gender) and disc herniation levels were determined and recorded for each patient. The pain intensities were defined with visual analog scale (VAS) scores. VAS is a subjective, yet valid, estimation of acute and chronic pain. Scores are recorded by patients’ marking on a 10 cm line that indicates a continuum between “no pain” on the far left and “the most intense pain” on the far right. VAS scores were determined preoperatively, immediately postoperatively, and after three and six months postoperatively. Pfirrmann's grading of the disease extent was determined on routine T2-weighted MRI sequence investigations. The Pfirrmann grading for disc disease is as follows: Grade I-a normally appearing disc; Grade II-disc with an inhomogeneous structure with a clear distinction between the nucleus pulposus and annulus fibrosus with normal height; Grade III-a gray, inhomogeneous disc with loss of a clear border between the nucleus pulposus and annulus fibrosus with a slightly decreased height; Grade IV-a dark gray, inhomogeneous and hypointense disc with significant loss of height; Grade V-a black inhomogeneous disc with the complete collapse of disc space. As previously stated, patients with Pfirrmann Grade V disc degeneration were excluded from our study.

Surgical procedure and specimen collection

The first author of this study (Mahmut Ozden) performed all surgical procedures at a single institution (Memorial Hospital, Istanbul). A simple single-level lumbar microdiscectomy was performed on all patients. Microdiscectomy was performed after partial hemilaminectomy, flavectomy, and root decompression with foraminotomy under surgical microscopy.

Immunohistopathological evaluation and quantitative/semi-quantitative analysis

CD68 was stained with an anti-CD68 primary antibody (KP1, DAKO). CD34 was stained with an anti-CD34 primary antibody (QBEnd10, DAKO). P53 was stained with an anti-CDP53 primary antibody (DO-7, DAKO). Lastly, brachyury was stained with mouse anti-Brachury/Bry/T-antibody (A4)/(A4, Medaysis). CD34 and CD68 positive cell counts were semi-quantitatively (as 0: no staining, 1: mild staining, 2: moderate staining, 3: diffuse staining, 4: intense staining) determined in 10 different high magnification areas, while brachyury expression was evaluated as present or absent since only slight and homogenous staining (n = 16) or complete lack of staining (n = 16) was observed among the assessed specimens. Countings and statistical evaluations were not performed for P53 as its staining was very rare and sparse.

Statistical analysis

The statistical program SPSS 20.0 (IBM, USA) was employed for statistical analysis. Descriptive statistical methods (i.e., mean, standard deviation [STD], median, minimum, maximum, and percentage) were utilized to evaluate the study data. Spearman correlation analysis was used to evaluate the relationship between quantitative variables including pathological (CD34, CD68) and clinical (age, pain densities, Pfirrmann grades) scale scores. Non-parametric Mann-Whitney U test was used to find any association between brachyury protein expression and other clinico-pathological features. Statistical significance was set at P < 0.05.

## Results

Herniation levels on L3-L4, L4-L5 and L5-S1 were encountered in four (12.5%), 16 (50%) and 12 (37.5%) patients respectively. Table 1 summarizes the demographic features and pain levels of the study population. There were equal numbers of male (n = 16) and female (n = 16) patients in the study with a median age of 42 years (min-max: 28-72). VAS values were given as mean ± STD in Table 1. Median values for VAS were 9 (min-max: 8-10), 1.5 (min-max: 0-3), 1 (min-max: 0-3), and 1 (min-max: 0-4) in preoperative, immediate postoperative, three months postoperative, and six months postoperative time intervals, respectively.

**Table 1:** Patient demographics and pain density (VAS scores) in disc hernia patients STD: Standard deviation, pre-op: preoperative, post-op: postoperative. VAS: Visual Analog Scale

Patient demographics		n (%)
Sex	Male	16 (50%)
	Female	16 (50%)
Age	Mean±Std	43.75±10.64
	Median (Min-Max)	42 (28-72)
Patient Pain		Mean±STD
VAS Scores	Pre-Op	8.97±0.74
	Post-Op/Immediate	1.5±1.02
	Post-Op/3th Month	1.13±0.87
	Post-Op/6th Month	1.22±1.26

**Figure 1:**
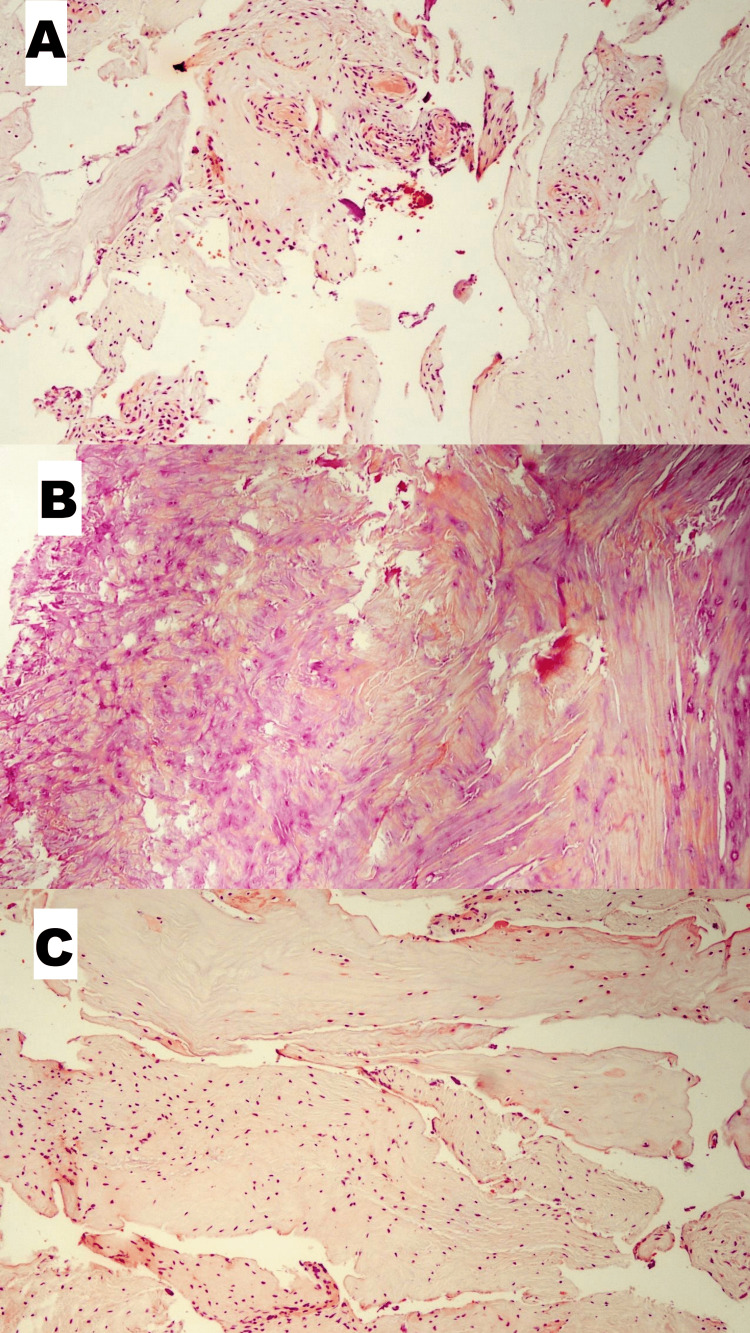
Classical immunohistopathological features of the degenerated disc tissues (A) Increased vascular density and histiocytic infiltration area in degenerated cartilage tissue (H&E, x100 magnification). (B) Degenerated nucleus pulposus (H&E, x40 magnification). (C)  Degenerated cartilage tissue (H&E, x100 magnification).

Figure 1 depicts our observations obtained from conventional histopathological analyses (hematoxylin-eosin [H&E] staining). Increased vascular densities and histiocytic infiltration areas were encountered in degenerated cartilage tissue (Figure 1A). Frequent areas of degeneration in nucleus pulposus and disc cartilage (Figures 1B, 1C) were witnessed.

**Figure 2:**
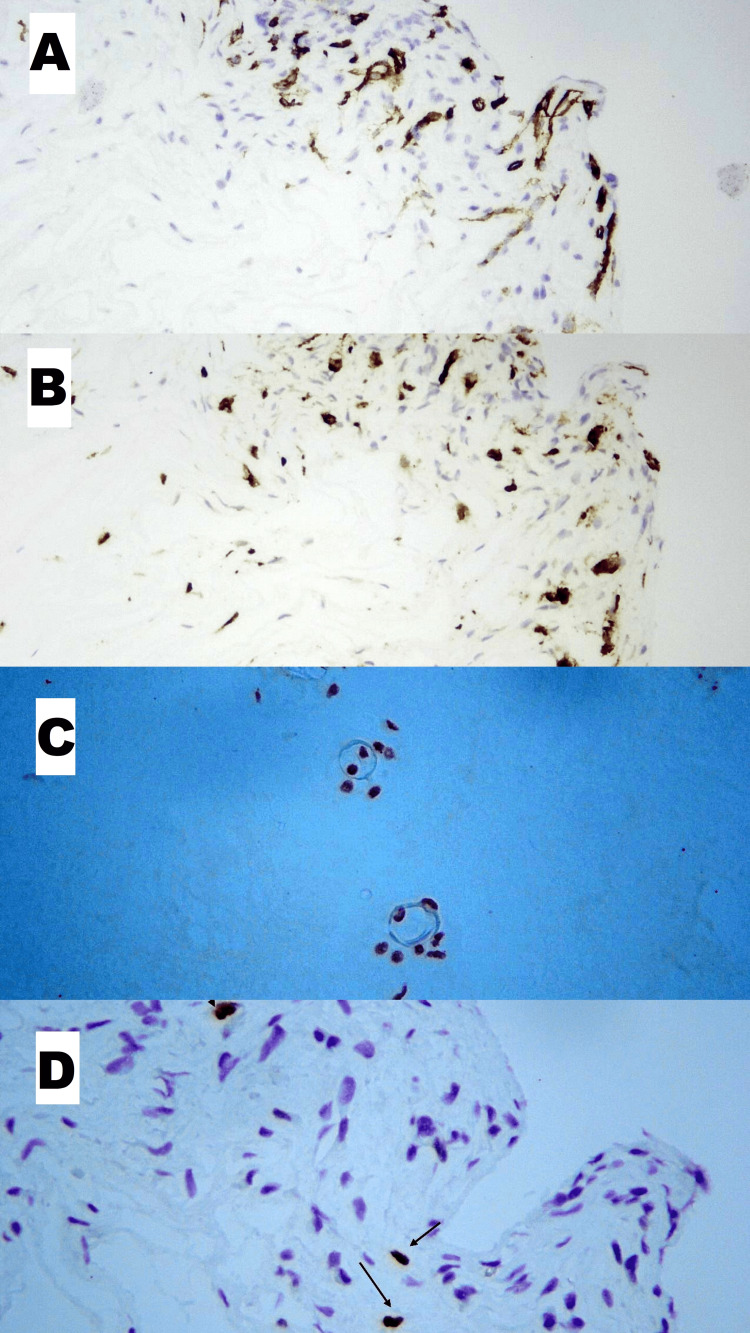
Immunohistopathological features of degenerated discs (A) CD34+ capillaries in areas of increased vascular density (H&E, x200 magnification). (B) CD68 positivity in histiocytic infiltration areas (x200 magnification). (C) Weak to moderate Brachury positivity in degenerated chondrocytes (x100 magnification). (D) Rare P53 positivity in the revascularized area (marked with arrows) (x400 magnification).

Figure 2 depicts our observations obtained from immunohistopathological analyses. Focal regions with higher vascular density stained with CD34 in microcapillaries were seen (Figure 2A). CD68 positivity in histiocytic infiltration areas was obvious (Figure 2B). Degenerated chondrocytes were sparsely stained with brachyury protein at weak to moderate intensities (Figure 2C). Peculiarly, weak areas of P53 staining were also seen around vascularization regions (arrows) (Figure 2D).

CD34 and CD68 staining intensities exerted a mild, albeit insignificant, positive correlation (R: 0.283, P = 0.116). There were also mild negative correlations between CD34 and VAS scores determined at the immediate postoperative and six months postoperative periods (R: -0.247 and -0.210, respectively). CD34 staining intensity significantly correlated with the Pfirrmann Grade (R = 0.604; P = 0.000). There was a mild negative correlation between CD68 and VAS scores determined at the immediate postoperative period (R: -0.248, P = 0.172) but not in other time periods. Patients age did not correlate with CD34 and CD68 staining intensities as well as with pain and Pfirrmann grading of the disease. CD68 staining intensity significantly correlated with the Pfirrmann grade (R = 0.526; P = 0.002). Pfirrmann grading negatively, albeit again insignificantly, correlated with VAS scores evaluated at the preoperative, immediate postoperative, and six months postoperative periods with R values equal to -0.204, - 0.291 and -0.192, respectively. Brachyury staining densities did not correlate with any of these parameters yet, the age tended to be higher in patients whose specimens were not stained with brachyury protein (mean age 47.1 ± 10.9 versus 40.4 ± 9.6 for patients who did not express and express brachyury protein respectively). Lastly, we did not encounter a noteworthy expression of P53 protein, except focal staining in two of the patients' specimens, which was very weak (only 2% of high magnification area was stained with immune dye against P53).

## Discussion

According to some investigators, pathological vertebral disc degeneration resembles the influence of aging on the discs. With aging, the nucleus pulposus loses proteoglycan, appears more collagenous and less hydrated, and the boundary between the nucleus and annulus becomes more indistinct. Boos et al. classified the pathological alterations in the degenerating intervertebral discs after analyzing lumbar specimens obtained from cadavers ranging from fetal to 88 years of age [7]. Their study revealed tears or formation of clefts, edge neovascularization and rim lesions, mucoid degeneration, and chondrocyte proliferation as the most frequent alterations in degenerated discs. Ammar et al. analyzed disc specimens from 21 patients with disc herniation according to aging-associated pathological disc changes classified previously by Boos et al. [1]. They reported that they found no significant pathological changes in herniated disc specimens, such as increased cartilage cell proliferation and clusters, within the nucleus pulposus. They also failed to find significant correlations between the major pathological changes of the extruded discs and weakness, paresthesia, and reflex changes [1]. Similar to the findings of Ammar et al., we also failed to show that inflammation and angiogenesis are correlated .with preoperative and postoperative pain levels in disc disease despite Pfirrmann grading scores correlating slightly negatively with VAS values. This latter observation may be explained by the denervation in later stages of disc degeneration where patients with highly degenerated discs paradoxically complain of lesser pain. There were also mild negative correlations between CD34 and VAS scores determined at the immediate postoperative and six months postoperative periods (R: -0.247 and R: -0.210, respectively) which may suggest that more angiogenic disc tissues are encountered in patients where denervation contributes to the clinical presentation.

In human disc tissues of fetuses, infants, and adolescents, CD68+ macrophages do not exist; however, they are found to be present in the nucleus pulposus of all subjects with morphologic signs of disc degeneration [8]. In an earlier study, Virri et al. examined CD68+ macrophages and blood vessel endothelia in disc tissues which were obtained from 20 disc hernia operations [9]. Both blood vessels and macrophages were found in 80% and 55% of the studied disc tissues respectively. Importantly, macrophages existed only in tissue regions with blood vessels and had likely infiltrated the tissue from them. The investigators hypothesized that some blood vessels were formed newly following tissue injury, whereas others existed in disc tissues before herniation. This was suggested due to the lack of an evident correlation between the existence of vessels and the preoperative pain duration [9]. In our view, this conclusion cannot be made easily, as pain intensity does not correlate with disease extent in disc herniation. Similarly, Habtemariam et al. found CD68+ macrophages in degenerated disc tissues, yet the abundance of these cells did not substantially differ in acute versus chronic disease [10]. Interestingly, Woertgen et al. found lesser postoperative VAS scores of pain in patients whose degenerated disc specimens harbored higher levels of CD68+ macrophages. They attributed these findings to a possible “foreign body reaction” against the extruded disc tissues [11]. We also found a mild negative correlation between the density of CD68+ macrophages and the intensity of pain during the early postoperative period. Despite this observation, we do not presume that a “foreign body reaction” can remove degenerated disc tissue debris so quickly as to alleviate the associated pain. Levels of proteolytic enzymes, such as aminopeptidases, increase in degenerated disc tissues [12]; hence, although very hypothetically, a major increase in proteolytic enzymes may cause the vanishing of innervating nerve fibers; thereby alleviating pain.

Koike et al. studied 20 disc specimens surgically excised from 17 patients with disc herniation (nine sequestration and 11 extrusion types) [13]. Many spindle-shaped cells positive for VEGF were found inside granulation tissue that invaded the cartilage matrix in a positive correlation with the number of CD34+ cells. CD68+ macrophages surrounded CD34+ cells that formed a lumen. Abundant CD68+ cells were observed with a positive correlation of CD34 cells. The investigators concluded that the VEGF produced by spindle-shaped cells induced neoangiogenesis in the granulation tissue that infiltrated the disc cartilage matrix, and that newly formed blood vessels acted as a passage for macrophages into the degenerated disc matrix [13]. The findings of our current study are parallel to the findings of Koike et al. as we also found a positive correlation between CD34+ and CD68+ cell intensities. A further finding of our study was that these cell intensities also positively correlated with Pfirrmann grading. It was also found that CD34+ endothelial cells positively correlated with pleiotrophin expression in degenerated human disc tissues [14]. Pleiotrophin binds to heparin and chondroitin/dermatan sulfate moieties and is highly present in developing fetal and neonatal tissues. In these tissues, pleiotrophin induces matrix synthesis and promotes blood vessel invasion into the hypertrophic region of the epiphyseal growth plate [14]. Hence, despite cellular senescence, some mechanisms have been proposed to cause disc degeneration processes. Alternative induction of regenerative pathways and embryonic protein reactivations may also be involved in the pathogenesis of the disease. Indeed, mild staining with brachyury was observed among the degenerated chondrocytes in our current study.

Djuric et al. investigated disc specimens and radiological MRI images of 119 patients with disc herniation [15]. They evaluated modic changes (MC), which are vertebral endplate signal changes encountered in subjects with lumbar disc herniation, and suggested that it was associated with the slower recovery rate in patients with disc herniation. In subjects demonstrating MC at baseline, patients became significantly more disabled if they showed substantial disc inflammation compared to patients with milder inflammation as assessed with CD68 staining [15]. Ford et al. also demonstrated that preoperative clinical signs of lumbar radiculopathy are more severe in patients whose surgically excised disc specimens harbored higher inflammatory changes [16]. Castro et al. analyzed pathological alterations in human annulus fibrosis (AF) failure by evaluating the herniated extracellular matrix (ECM) [17]. They performed an immunohistopathological analysis of 39 AF specimens and six scoliosis controls for inflammatory markers. They determined correlations between these markers in ECM and the degree of nucleus pulposus containment (contained, uncontained, or protruded) and the age of patients. Fibronectin staining densities and α-smooth muscle actin+ cells were increased in human annulus fibrosus while matrix metalloproteinase-12 (MMP12) CD68+ macrophage cell levels remained constant. These pathological changes witnessed in annulus fibrosis with herniation progression were independent of the age of patients [17]. We also failed to find any correlation of age with pathological and clinical parameters (pain, Pfirrmann grade); however, in contrast to the findings of Castro et al., we found that CD68+ intensities change in different gradings of disease.

One of the main mechanisms of tissue degeneration in pathological processes and aging is cellular senescence/aging, which means that cells reach their limit of regeneration. In this study, we aimed to examine whether disc degeneration was also associated with cellular senescence as assessed with brachyury and P53 protein staining densities. During development, disc tissues originate from the notochord which is regulated by brachyury protein [18-21]; hence, brachyury is a marker of cells of notochordal origin. While some earlier studies showed no correlation between brachyury protein expression and disc aging and degeneration, later studies reported decreases in brachyury expression in aging and degeneration [19,20]. Nonetheless, in contrast, we observed brachyury expressions, especially around the degenerating chondrocytes, indicating that whatever the cause of disc tissue stress, this process may induce molecular pathways related to regeneration. Lastly, we also determined P53, a protein mainly known for its tumor suppressive functions, and expression densities in disc samples [22]. TP53 gene product, P53 protein, functions as a “genomic and cellular gatekeeper” by ensuring that cellular progeny are protected from DNA damage that occured in ancestral cells [23]. Following P53 research that lasted for 43 years, it was recently revealed that P53 is also one of the essential regulators of cellular senescence [24]. Therefore, our final aim of the study was to define P53 expression in degenerated disc samples as there are sparse reports in this area [18,25]. Our findings regarding uncommon (6.25%) and very weak expressions of P53 in degenerated disc tissues contrasted with the findings of Kim et al., who found that all (100%, n = 25) degenerated human disc specimens that they studied expressed P53 [18]. Furthermore, our results were not parallel to preclinical disc degeneration models in rats which revealed P53 expression in 59% of cases with a coexpression rate of 53.8% of VEGF protein [26].

Future prospects, limitations, and strengths of the study

Our results confirmed that inflammation and angiogenesis simultaneously occur in disc herniation disease and add further data demonstrating that these factors correlate with disease extent as shown with Pfirrmann grading. Hemapoietic stem cells (HSC) also express CD34, but we think that HSCs are less likely to exist within the CD34+ areas which we observed due to: i) CD34 and CD68 stainings mostly overlap which is very logical as neoangiogenesis and inflammation mutually stimulate each other with paracrine signals and hence; the observed CD34+ cells represent endothelia; ii) despite HSCs are shown to possibly infiltrate inflammatory tissues including cancerous tissues, we think that they are less likely to invade cartilage tissue which possibly release much lesser systemic inflammatory and chemotactic molecules and their anatomy is less likely to be infiltrated by more delicate and sparse cells. Another limitation of our study is the semiquantification of markers of inflammation and angiogenesis. Conversely, our current study has relative strengths since a homogenous group of patients was used with equal number of male and female subjects and a single etiopathogenesis. Many other degenerative disorders were excluded, and lastly, pain levels were studied across three different time periods.

As a future prospect, our current study may indicate potential anti-angiogenic agents in degenerative disc disease. In clinical practice, anti-angiogenic medicines are successfully used in the treatment of cancer and proliferative retinopathy. Noteworthy, in a preclinical study published in 2022, it was shown that local delivery of bevacizumab alleviates disc degeneration and MMP3 production [27]. Systemic application of antiangiogenic agents may cause considerable side effects and they are very costly. However, with pharmaceutical technologies being developed every day, the cost of such agents will improve in the near future. In this context, coadministration of anti-inflammatory agents and drugs that selectively target pathological angiogenesis may reduce the need for surgery in some patients with disc herniation.

## Conclusions

Despite there exist studies that demonstrated the increased presence of macrophages and vessel endothelia in disc tissues of patients with lumbar disc hernia, to the best of our knowledge, there exist no investigations, on whether their intensity correlates with the clinical disease severity as assessed with Pfirrmann grade. Further, in this clinico-pathological study, we also determined pain levels by VAS scores and determined their association with Pfirrmann grades. Peculiarly, a negative correlation, albeit statistically insignificant, was observed between VAS scores and Pfirrmann grades. This can be attributed to denervation, where patients with large, protruded discs and suffered from a prolonged period of disc hernia experience paradoxical relief from pain. We did encounter a slight, yet detectable expression of brachyury protein in degenerated discs which may be explicable with a compensatory regeneration attempt accompanying disc injury. Lastly, p53 expression in degenerated disc tissues was negligible, which is opposite to the published research suggesting that it may play a role in disc hernia disease. Our current results confirm the presence and association of inflammation and angiogenesis in herniated discs and further suggest their correlation with the extent of the disease. Pathological analysis of herniated disc tissues will pave the way for the development of future medical management strategies in degenerative disc diseases.
